# Cardiorespiratory Responses to Glittre ADL Test in Bronchiectasis: A Cross-Sectional Study

**DOI:** 10.1155/2018/7470387

**Published:** 2018-12-17

**Authors:** Ravoori Hena, Gopala Krishna Alaparthi, K. Shyam Krishnan, R. Anand, Vishak Acharya, Pretham Acharya

**Affiliations:** ^1^Department of Physiotherapy, Kasturba Medical College, Manipal Academy of Higher Education, Bejai, Mangaluru 575004, India; ^2^Associate Professor, Department of Physiotherapy, Kasturba Medical College, Manipal Academy of Higher Education, Bejai, Mangaluru 575004, India; ^3^Asst. Professor, Senior Scale, Department of Physiotherapy, Kasturba Medical College, Manipal Academy of Higher Education, Bejai, Mangaluru 575004, India; ^4^Professor, Department of Pulmonary Medicine, Kasturba Medical College, Manipal Academy of Higher Education, Mangaluru 575004, India; ^5^Professor, Department of Pulmonary Medicine, Kasturba Medical College, Manipal Academy of Higher Education, Mangaluru 575004, India; ^6^Associate Professor, Department of Pulmonary Medicine, Kasturba Medical College, Manipal Academy of Higher Education, Mangaluru 575004, India

## Abstract

**Background:**

Bronchiectasis is a chronic respiratory condition characterised by chronic sputum production, fatigue, and dyspnoea. These symptoms will lead to reduced exercise capacity and a reduced ability to carry out activities of daily living. Glittre ADL test is a valid and reliable test which evaluates the activities of daily living.

**Aim:**

To investigate whether the Glittre ADL test can differentiate the functional capacity and cardiorespiratory responses of patients with bronchiectasis from those healthy individuals using the six-minute test as a functional performance standard.

**Methods:**

This study included 30 subjects: 15 bronchiectasis and 15 age- and gender-matched healthy subjects. The patients and healthy subjects were made to perform the Glittre ADL and six-minute test on two consecutive days. Parameters such as time taken, distance walked, HR, RR, SpO_2_, and dyspnoea were recorded before and after the tests.

**Results:**

The performance of bronchiectasis was worse than the healthy group on the Glittre ADL test (4.78 ± 1.33 min, 3.94 ± 0.82 min, *p*=0.04). Distance walked in the six-minute walk test by the bronchiectasis was 42 meters lesser than the healthy (400.33 ± 77.99, 442 ± 89.21, *p*=0.18). The Glittre ADL test was correlated with 6MWT when the total sample was analysed (*r*=−0.41, *p*=0.05). There was moderate positive correlation between heart rate variation, dyspnoea, respiratory rate, and peripheral saturation (SpO_2_) between the tests (Glittre heart rate versus six-minute walk test heart rate (*r*=0.55, *p*=0.001); Glittre (Borg) versus six-minute walk test (Borg) (*r*=0.72, *p*=0.00); Glittre respiratory rate versus six-minute walk test RR (*r*=0.62, *p*=0.00); Glittre SpO_2_ versus six-minute walk test SpO_2_ (*r*=0.40, *p*=0.02)). The bronchiectasis group had a statistically significant higher (*p*=0.08, *p*=0.46) increase in dyspnoea and RR than the controls in both the Glittre ADL test and six-minute walk test (*p*=0.009, *p*=0.03), with the similar HR variation in both the groups (*p* > 0.05). There was statistical difference in peripheral oxygen saturation in bronchiectasis in the six-minute walk test (*p*=0.03).

**Conclusion:**

The Glittre ADL test induced similar cardiorespiratory responses when compared to the six-minute walk test. So, the Glittre ADL test can be used as an assessment tool besides the six-minute walk test for the more complete evaluation of functional capacity and activities of daily living.

## 1. Introduction

Bronchiectasis is a persistent or progressive condition characterised by dilated thick-walled bronchi. It exhibits recurrent or prolonged bronchial infection related to irreversibly damaged bronchi, with the symptoms including cough, wheeze, sputum production, dyspnoea, and reduced functional capacity [1, 2]. Functional capacity often deteriorates with time, despite adequate medical interventions, such as antibiotic treatment and bronchodilators [3]. Reduced functional capacity in bronchiectasis is multifactorial, i.e., altered pulmonary mechanics, inefficient gas exchange, decreased muscle mass, and confounding psychological morbidity lead to a progressive detraining effect [1, 2].

Functional capacity and limitations in performing activities of daily living (ADL) can be assessed through questionnaires and field tests [4]. However, self-reported questionnaires can be influenced by psychological factors, cognitive alterations, or adoption of a sedentary lifestyle [5], and they are often subject to recall bias, which does not provide accurate functional capacity and accurate estimation of free-living energy expenditure [6, 7].

The six-minute walk test is one of the field tests which is widely used measure of functional status and predictor of prognosis across a multitude of respiratory conditions due to the ease of implementation, low cost, and representativeness of daily activities. It evaluates the general functional responses of the pulmonary, cardiovascular, and muscular system to evaluate functional status of patients with bronchiectasis. However, this test only evaluates the capacity of an individual to move, and it ignores the arms in the execution of many activities of daily living [8–10].

Skumlien et al. developed Glittre activities of daily living (Glittre ADL test) to evaluate the functional status and capacity to carry out activities of daily living in COPD [11]. The modified version of pulmonary functional status and dyspnoea questionnaire (PFSDQ-M) was based on the four relevant questions, and the London Chest Activity of Daily Living (LCADL) scale includes activities of daily living such as walking, climbing stairs, and performing activities using upper extremities [12, 13].

Glittre ADL may be considered more complete than the six-minute walk test for evaluating COPD individuals, as it involves activities such as sitting, standing, walking on the horizontal flat ground, ascending and descending steps, and shifting the objects from shelves along with arm and trunk movements, where functional capacity is more compromised compared to the six-minute walk test which involves only walking. In the pulmonary rehabilitation program, the six-minute walk test is reported to be responsive, reproducible, quick, and easy to apply in hospital setting [11, 14].

There is a lack of retrievable literature in the comparison with Glittre ADL performance between bronchiectasis and healthy subjects. The present study aimed at investigating whether the Glittre ADL test can differentiate the functional capacity and cardiorespiratory response of patients with bronchiectasis from those healthy individuals using 6MWT as a functional performance standard.

## 2. Methodology

### 2.1. Inclusion Criteria

- Patients with stable bronchiectasis referred by pulmonologist or physician
- Age- and gender-matched subjects recruited from the community for the healthy group

### 2.2. Exclusion Criteria

- History of pulmonary conditions such as TB and asthma
- History of musculoskeletal conditions such as severe osteoarthritis or osteoporosis, recent fractures
- History of obesity, cancer, and uncontrolled diabetes mellitus
- History of recurrent haemoptysis
- History of acute myocardial infarction
- Subjects who were on oxygen therapy
- Users of orthopaedic orthosis or prosthesis
- Subjects who are unable to perform the test
- Subjects whose symptoms exacerbate during the data collection

## 3. Study Procedure

The study was carried out in the Kasturba Medical College Hospitals, Mangalore, over a period of one year starting from March 2017 to March 2018. The study as approved by the Institutional Ethics Committee (IEC KMC MLR 11-16/296) of the Kasturba Medical College, Mangalore. Eligible patients were selected based on the inclusion and exclusion criteria. The purpose of the study was made clear to each patient, and a written informed consent was obtained prior to involving them in the study. Subjects with bronchiectasis who were referred by the pulmonologist and healthy subjects who were willing to participate recruited from the community. Each patient underwent a formal evaluation of the pulmonary function test according to ATS/ERS. Later, Glitter ADL test and six-minute walk test were assessed separately in each group for all individuals.

### 3.1. Description of Glittre ADL Test

The Glittre ADL test consists of a standardized 10-meter circuit, where the individual was instructed to go through the following sequence of activities in the shortest time. The individual stands up from a sitting position and walks on flat ground; halfway through the circuit, the individual climbs up and then down a pair of stairs (17 cm high × 27 cm deep each step) and walks on flat ground again.

At the end of the circuit, there is a bookshelf where the individual has to move three 1 kg objects from the top shelf (at shoulder height) one at a time onto the bottom shelf (at waist height) and subsequently onto the floor; then, the objects have to be moved again onto the bottom shelf and, finally, returned to the top shelf; next up the individual returns to their initial position. Immediately afterwards, the individual starts another lap, going through the same ADL circuit. In order for the test to be considered concluded, the individual has to complete 5 laps. During the test, the individual has to carry a backpack containing 2.5 kg (women) and 5 kg (men) [11]. The subjects were instructed to complete the test as quickly as possible, and they were allowed to rest during the test, but were requested to resume the test as soon as possible. The subjects were not encouraged during the test.

Heart rate (HR), respiratory rate (RR), and peripheral oxygen saturation (SpO_2_) were measured by using the pulse oximeter, the modified Borg scale is used to measure dyspnoea at the beginning and immediately after the test. The main parameter was the total time required for the completion of the test. The longer it takes a patient to perform the test, the worst their functional capacity. Two tests were performed and separated by at least 30 minutes of rest, and the best performance was considered.

### 3.2. Description of Six-Minute Walk Test

The 6MWT was performed by all subjects in a 30-meter indoor hospital corridor, under the supervision of a physiotherapist, according to the ATS guidelines [15]. Participants were instructed to walk as far as possible, with the aim of achieving their maximum possible walking distance in six minutes. Standardized instructions were provided each minute. Participants were permitted to stop and rest during the test but were instructed to resume walking as soon as they were able to.

Heart rate (HR), respiratory rate (RR), and oxygen saturation (SpO_2_) were measured using the pulse oximeter, and patients rated the magnitude of their perceived breathlessness using a modified Borg scale (0–10 points) at the beginning and immediately after the test. The outcome of the test was the covered distance; the longer the distance covered by the patient, the better their functional capacity. Two tests were performed, separated by at least 30 minutes of rest, and the best performance was considered.

#### 3.2.1. Sample Size

The sample size was calculated based on the Glittre ADL time in a pilot study (3 subjects in both groups). The following formula was used for calculation:

(1) $$\begin{array}{c} N=\frac{2\left(Z\alpha +Z\beta \right)2\left(1+\left(n-1\right)\sigma \right)}{n{\left(\left(\mu_{1}-\mu_{2}\right)/\sigma \right)}^{2}}, \end{array}$$

where *N* is the number of subjects in each of two groups, *σ*^2^ is the assumed common variance in the two groups, *µ*_1_ − *µ*_2_ is the difference in means of the two groups, *n* is the number of time points, and *p* is the assumed correlation of the repeated measures. Standard deviation for the change scores among bronchiectasis patients was 20.8 sec (*µ*_1_) and among normal subjects was 11.5 sec (*μ*_2_); mean difference between them for the change scores was 20.3 sec; *α* error was 5%, with 90% power, and adding 10% nonresponse error, the total sample size came to 15 in each group.

#### 3.2.2. Data Analysis

Data were analysed using SPSS (V 17.0). Within the group, analysis was done using the Wilcoxon signed-rank test, and between the group, analysis was done by the Mann–Whitney *U* test. The Karl Pearson correlation and coefficient was used to correlate variables between the tests.

## 4. Results

A total 34 subjects were selected who matched inclusion criteria in the study, of which 30 subjects were included in the study. From 34 subjects selected to participate in this study, 4 subjects were excluded as follows: 1 had fear of fall during stair climbing, 2 exacerbated during the evaluation period, and 1 got discharged after the six-minute walk test. Thus, 30 patients concluded the study. In each group, 15 bronchiectasis and 15 age- and gender-matched healthy subjects performed two Glittre ADL tests and two six-minute walk tests on two consecutive days.

Table 1 summarises the demographic characteristics and baseline data of the bronchiectasis and healthy subjects. The groups in both the tests did not differ in age, height, and heart rate (*p*>0.05) but showed difference in weight, basal metabolic rate, respiratory rate, dyspnoea, and SpO_2_ (*p* < 0.05).  Table 2 shows the comparison of the functional capacity and postcardiorespiratory responses of bronchiectasis and healthy subjects in the Glittre ADL test and six-minute walk test. The bronchiectasis group spent 2 min more time than the healthy group in the Glittre ADL test which was statistically significant (*p*=0.04) and walked 42 meters lesser than healthy in the six-minute walk test which was not statistically significant (*p*=0.18).

Heart rate behaviour was similar in both the groups during the Glittre ADL test (*p*=0.71) and six-minute walk test (*p*=0.93), demonstrating a physiological increase. There was significant increase in the respiratory rate in the bronchiectasis group when compared to the healthy group in both the tests (*p*=0.09, *p*=0.03). The bronchiectasis group showed decreased SpO_2_ in the six-minute walk test when compared to the healthy group (*p*=0.03). The bronchiectasis group had showed increase in dyspnoea in both the tests (*p*=0.003, *p*=0.05) when compared to healthy subjects.

**Figure 1:**
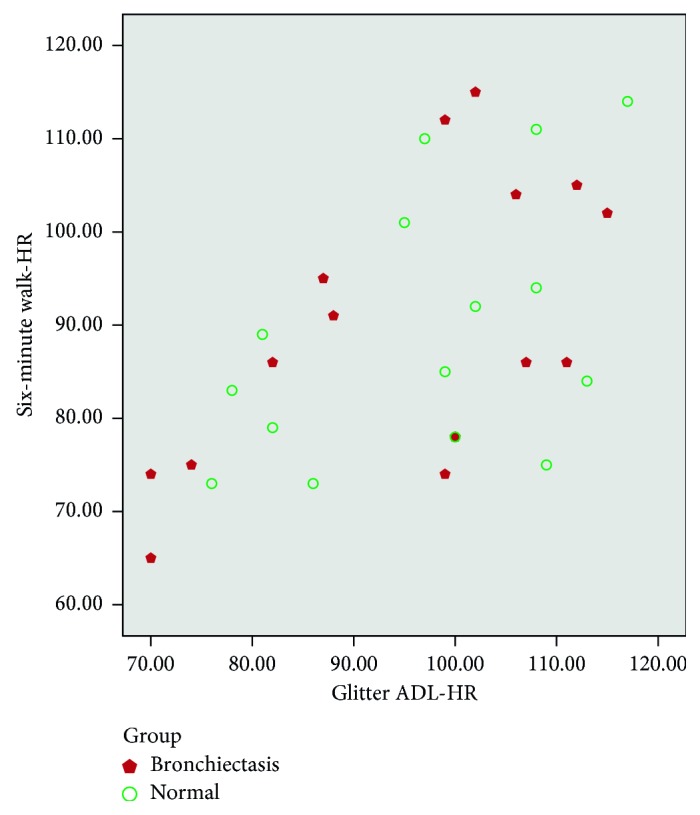
Correlation between Glittre ADL test HR (heart rate) and six-minute walk test HR (heart rate).

**Figure 2:**
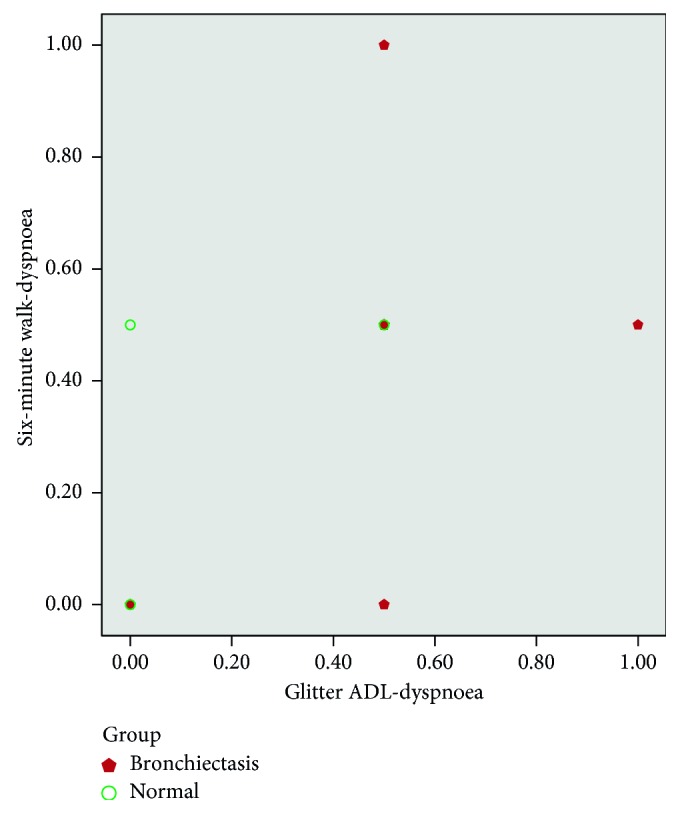
Correlation between six-minute walk test Borg dyspnoea and Glittre ADL test Borg dyspnoea.

**Figure 3:**
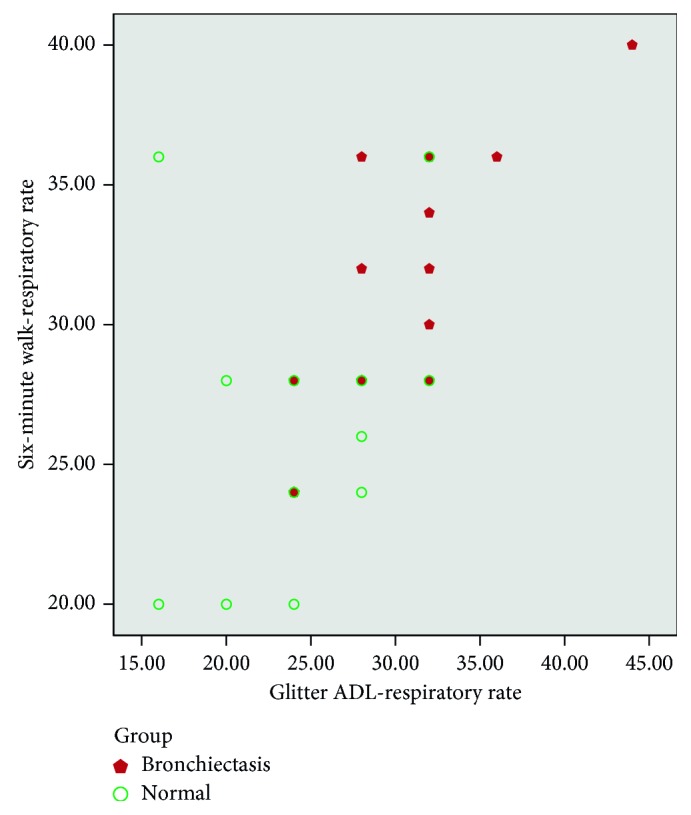
Correlation between six-minute walk test RR (respiratory rate) and Glittre ADL test RR (respiratory rate).

**Figure 4:**
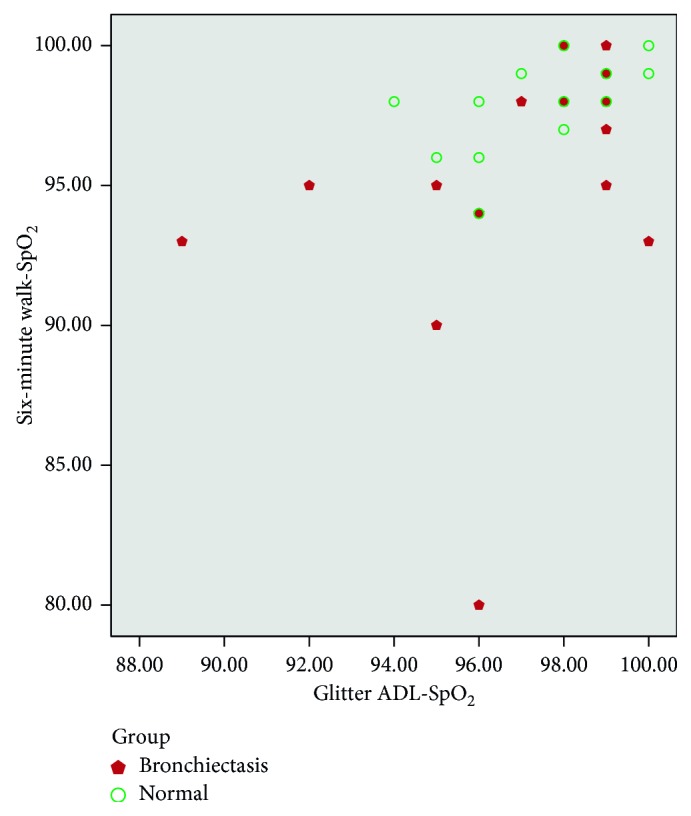
Correlation between six-minute walk test SpO_2_ (peripheral oxygen saturation) and Glittre ADL test SpO_2_ (peripheral oxygen saturation).

**Figure 5:**
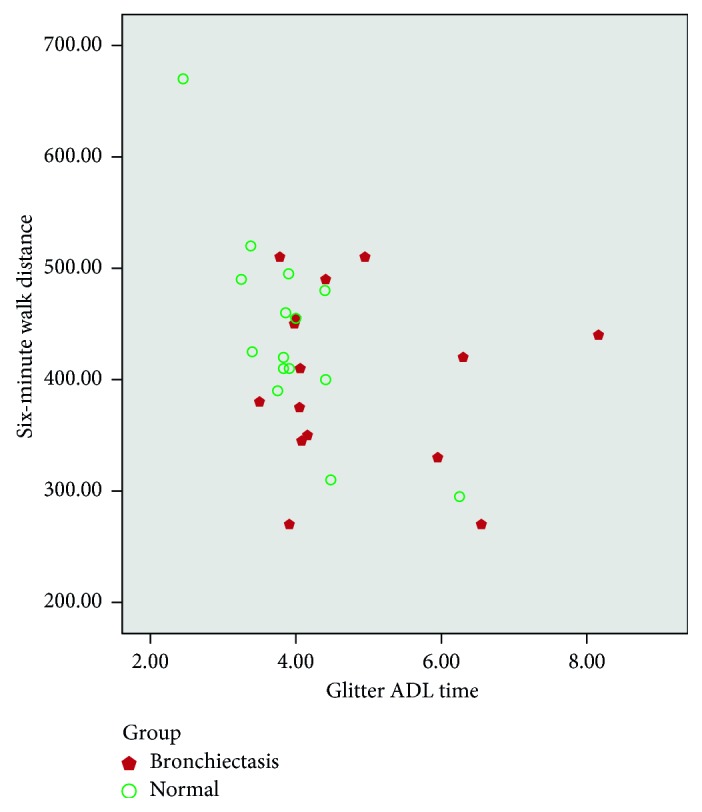
Correlation between Glittre ADL time and six-minute walk distance.

There was moderate correlation between heart rate variation, Borg dyspnoea, respiratory rate, and peripheral saturation (SpO_2_) between the tests (Glitter heart rate versus six-minute walk test heart rate (*r*=0.55, *p* ≤ 0.01; Figure 1); Glittre (Borg) versus six-minute walk test (Borg) (*r*=0.72, *p* ≤ 0.01; Figure 2); Glittre respiratory rate versus six-minute walk test RR (*r*=0.62, *p* ≤ 0.01; Figure 3); Glittre SpO_2_ versus six-minute walk test SpO_2_ (*r*=0.40, *p*=0.02; Figure 4)). There was moderate negative correlation between Glittre ADL test time and six-minute walk test distance (Glittre functional capacity (FC) versus six-minute walk test functional capacity (FC) (*r*=−0.41, *p*=0.02; Figure 5).

## 5. Discussion

The current study investigated whether the Glitter ADL test can distinguish the functional capacity and cardiorespiratory responses in patients with bronchiectasis from healthy individuals. Our study results showed that the Glittre ADL test can differentiate between bronchiectasis and healthy subjects. On an average, it was found that bronchiectasis subjects took 2 minutes longer to complete the test when compared to age- and gender-matched healthy subjects. Bronchiectasis group walked 42 meters lesser than the healthy subjects which show that their functional capacity was decreased when compared to the healthy group.

In the current study, the Glittre ADL test was able to discriminate the group who walked lesser distance in the six-minute walk test. Similar to the results found in a study by Corrêa et al. [16], the results showed that bronchiectasis subjects took 4.7 ± 1.33 minutes to complete the Glittre ADL test whereas the healthy subjects took 3.94 ± 0.82 minutes. The decreased level of performance was due to the presence of pulmonary dysfunction which is progressive airflow obstruction. The strong relationship was found between dyspnoea and functional capacity as expiratory flow is limited due to dynamic hyperinflation leading to reduced tidal volumes and work of breathing [10, 17]. Extrapulmonary features such as skeletal muscle abnormalities (respiratory and limb) caused by inflammation, gas exchange abnormalities, electrolyte imbalance, inactivity, malnutrition, and drugs in addition to respiratory tract involvement which negatively affect the exercise capacity and perception of fatigue are more common in bronchiectasis [18].

In our study, there is significant difference between bronchiectasis and healthy subjects in both weight and body mass index (BMI) which shows that the bronchiectasis group has lesser body weight and lower BMI than the age- and gender-matched healthy subjects; these findings are because bronchiectasis exhibit weight loss and nutrition depletion. A study by Cano et al. found that the prevalence of malnutrition (defined as a BMI of <20 kg/m^2^) was nearly 30% among patients with bronchiectasis. A poor nutritional status (malnutrition) is directly related to decreasing pulmonary function which negatively affects the exercise capacity is one of the reasons why there is declined functional performance in bronchiectasis than the healthy subjects.

The studies performed on other population with the cardiovascular and pulmonary pathologies show similar results. It has found in a study by Valadares et al. [19] that subjects with heart failure took 6.3 ± 4.8 minutes to complete the test, whereas another study by Fernandes-Andrade et al. showed that subjects with cardiovascular disease took 3.24 minutes to complete [20]. Studies on application of the Glittre ADL test on COPD patients have shown varying results. Corrêa et al. found that COPD patients took an average time of 5.26 ± 2.9 minutes to complete the Glittre ADL test, whereas Karloh et al. [21] and Tufanin et al. found the time to be 4 minutes. This inequality has occurred due to the different severity or disease in which different mechanism have adverse effect on pulmonary function which will affect the activities of daily living [22].

In addition, the cardiorespiratory response, heart rate, was similar in both the tests. Respiratory rate and dyspnoea were found to be more in bronchiectasis group than the healthy group in both the tests. In the Glitter ADL test, there was greater increase in respiratory rate and dyspnoea in bronchiectasis when compared to the bronchiectasis group in the six-minute walk test. It could be due to multiple tasks which were included in the glitter ADL test as the different tasks require different energy demand.

The study by Cavalheri et al. compared the energy expenditure in 5 ADL based on the Glittre test and found that walking up and down stairs induced greater energy expenditure, dyspnoea, and fatigue in patients with pulmonary pathologies [23]. Gulrat AA et al. measured physiological responses of multiple tasks which are in the Glittre ADL test and found that Glittre walking and Glittre shelf resulted in similar physiological load, the only task that induced a ventilatory demand similar to the Glittre ADL was the Glittre shelf. It supports the result found in our study that the respiratory rate and dyspnoea were greater in the Glittre ADL bronchiectasis group than the six-minute walk test because the Glittre ADL test included the task performed by the unsupported arm [24].

In our study, the baseline peripheral oxygen saturation of the bronchiectasis group was less when compared to the healthy group in both the tests. There was 2% decrease in oxygen saturation which was statistically significant when compared to the control group (*p* ≤ 0.01). This result shows the presence of exercise-induced desaturation, which is the characteristic of chronic pulmonary disease such as bronchiectasis. The study by Hsieh et al. showed lowest oxygen desaturation values during the six-minute walk test in bronchiectasis. The six-minute walk test induced decrease of peripheral oxygen in the bronchiectasis group when compared to the Glitter ADL test bronchiectasis group, it could be due to the sitting task in the Glitter ADL where the patients sit on the chair to restart another round, and there is a reduction in metabolic and ventilator overload [9].

Our study showed moderate positive correlation between heart rate variation, dyspnoea, respiratory rate, and peripheral saturation (SpO_2_) between the tests (Glitter heart rate versus six-minute walk test heart rate (*r*=0.55, *p* ≤ 0.01); Glittre (Borg) versus six-minute walk test (Borg) (*r*=0.72, *p* ≤ 0.01); Glittre respiratory rate versus six-minute walk test RR (*r*=0.62, *p* ≤ 0.01); Glittre SpO_2_ versus six-minute walk test SpO_2_ (*r*=0.40, *p*=0.02)), whereas Glittre ADL time and six-minute walk test were correlated with the present study which showed moderate negative correlation (*r*=−0.41, *p*=0.02).

This is similar to the studies which were done by Skumlien et al. [11] and Corrêa et al. [16] on COPD (*r*=−0.82, *p* < 0.05, *r*=−0.64, *p* < 0.05). This correlation summarised that the bronchiectasis group took longer time to complete the Glittre ADL test and walked lesser distance, whereas healthy individuals took lesser time to complete Glittre ADL test and walk larger distance in the six-minute walk test. However, important associations were observed between the variables.

Limitations of the study are as follows: the participated bronchiectasis were mostly with mild to moderate disease. No questionnaires were used for either bronchiectasis group or healthy group to evaluate their physical activity in this study. The nonhomogeneity (body weight) of the groups in our study is one of the limiting factors.

Future studies can be carried out to evaluate functional capacity, cardiac, ventilator, and metabolic responses in the bronchiectasis during the Glittre ADL test using larger sample size. Further studies can be carried out using the Glittre ADL test as an evaluating tool in various cardiac and pulmonary conditions to evaluate the functional capacity.

## 6. Conclusion

The Glittre ADL test induced similar cardiorespiratory responses when compared to the six-minute walk test. So, the Glittre ADL test can be used as an assessment tool besides the six-minute walk test for the more complete evaluation of functional capacity and activities of daily living.

## Figures and Tables

**Table 1:** Demographic characteristics.

Characteristics	Bronchiectasis	Healthy	*p* value
	(*N*=15)	(*N*=15)	(<0.05)
Age (years) (mean ± SD)	50.8 ± 11.55	50.73 ± 11.47	0.97
Gender (*n*), M : F	3 : 12	3 : 12	—
Height (cm)	152.0 ± 11.05	151.40 ± 14.18	0.89
Weight (kg) (mean ± SD)	46.53 ± 11.10	53.47 ± 5.975	0.04
BMI (mean ± SD)	20.12 ± 4.03	23.62 ± 3.35	0.01
*Pulmonary function test*			
FVC (L)	1.98 ± 0.43	2.55 ± 0.44	0.01
FEV1 (L)	1.35 ± 0.68	2.17 ± 0.63	0.01
FEV1/FVC	68.18 ± 9.6	80.9 ± 11.9	0.01
*Glittre ADL test (mean* ± *SD)*			
HR	81.93 ± 12.71	80.13 ± 12.37	0.69
RR	26.93 ± 4.65	21.07 ± 4.13	0.01
SpO_2_	96.80 ± 2.21	98.27 ± 1.16	0.03
Borg	0.23 ± 0.26	0.00 ± 0.00	0.03
*Six-minute walk test (mean* ± *SD)*			
HR	79.87 ± 18.51	81.13 ± 10.15	0.81
RR	26.93 ± 3.84	21.33 ± 3.90	0.01
SpO_2_	97.07 ± 1.33	98.20 ± 1.15	0.01
Borg dyspnoea	0.27 ± 0.26	0.00 ± 0.00	0.01

cm, centimetre; BMI, basal metabolic rate; HR, heart rate; RR, respiratory rate; SpO_2_, peripheral oxygen saturation.

**Table 2:** Comparison of functional capacity and postcardiorespiratory responses of bronchiectasis and healthy subjects in Glittre ADL test and six-minute walk test.

Variables	Bronchiectasis (mean ± SD)	Healthy (mean ± SD)	Difference	*p*
*Glittre ADL test*				
Time (minute)	4.78 ± 1.33	3.94 ± 0.82	2.09	0.04
HR	94.80 ± 15.33	96.73 ± 13.33	−0.36	0.71
RR	29.87 ± 5.42	24.53 ± 4.98	2.80	0.09
SpO_2_	96.73 ± 3.03	97.60 ± 1.84	−0.94	0.35
Borg dyspnoea	0.47 ± 0.23	0.17 ± 0.24	−2.98	0.03
*Six-minute walk test*				
Distance (meters)	400.33 ± 77.99	442 ± 89.21	−1.36	0.18
HR	89.87 ± 15.24	89.40 ± 13.97	0.08	0.93
RR	30.67 ± 5	26.53 ± 4.93	2.28	0.03
SpO_2_	95 ± 5.04	98 ± 1.65	−2.19	0.03
Borg dyspnoea	0.40 ± 0.28	0.20 ± 0.25	−1.91	0.05

HR, heart rate; RR, respiratory rate; SpO_2_, peripheral oxygenation.

## Data Availability

The data used to support the findings of this study are included in the article.
